# Building Multiple Classifier Systems Using Linear Combinations of Reduced Graphs

**DOI:** 10.1007/s42979-023-02194-1

**Published:** 2023-09-27

**Authors:** Anthony Gillioz, Kaspar Riesen

**Affiliations:** 1 Institute of Computer Science, University of Bern, Neubrückstrasse 10, 3012 Bern, Switzerland; 2 Institute for Informations Systems, University of Appl. Sci. and Arts Northwestern Switzerland, 4600 Olten, Switzerland

**Keywords:** Structural pattern recognition, Graph matching, Genetic algorithm, Multiple classifier systems

## Abstract

Despite great efforts done in research in the last decades, the classification of general graphs, i.e., graphs with unconstrained labeling and structure, remains a challenging task. Due to the inherent relational structure of graphs it is difficult, or even impossible, to apply standard pattern recognition methods to graphs to achieve high recognition accuracies. Common methods to solve the non-trivial problem of graph classification employ graph matching in conjunction with a distance-based classifier or a kernel machine. In the present paper, we address the specific task of graph classification by means of a novel framework that uses information acquired from a broad range of reduced graph subspaces. Our novel approach can be roughly divided into three successive steps. In the first step, differently reduced graphs are created out of the original graphs relying on node centrality measures. In the second step, we compute the graph edit distance between each reduced graph and all the other graphs of the corresponding graph subspace. Finally, we linearly combine the distances in the third step and feed them into a distance-based classifier to obtain the final classification result. On six graph data sets, we empirically confirm that the proposed multiple classifier system directly benefits from the combined distances computed in the various graph subspaces.

## Introduction

A *graph*, in its generic formulation, is a universal representational formalism that consists of a finite set of basic entities (termed *nodes*), in addition to a set of *edges* that might exist between pairs of nodes. The flexibility and expressiveness of graphs lies, on one hand, in the set-theoretic definition of nodes and, on the other hand—and maybe more importantly—in the possibility of modeling relationships via edges.

Due to their great generality and flexibility, graphs can be used to learn and study data from a broad variety of applications (in particular in pattern recognition and related fields [1]). Prominent examples of classes of objects, which can be formally represented in a suitable and natural way by means of graphs, are molecular compounds [2], protein structures [3], binary executables [4], or networks [5], to name just a few examples (see [6] for more examples that emphasize the universality of this specific data structure).

When the underlying data in a pattern recognition scenario consists of both features along with relationships that might exist between different subparts of the data, we consider this to be structural pattern recognition. Such data are best formalized by means of graphs or trees. This is in stark contrast to statistical pattern recognition that employs quantitative features that are encoded in *n*-dimensional feature vectors. Both approaches share common roots that consists of extracting efficient representations out of a given set of data and mapping the extracted representations to one of the possible classes. Unfortunately, the inherent relational structure of graphs makes it impossible to directly translate the methods, formerly proposed for statistical data, to graph or tree data.

A popular strategy to tackle this issue is to map graphs into an implicit or explicit embedding space and eventually apply statistical pattern recognition algorithms to the embedded graphs. However, it is a largely unresolved problem to find an embedding that respects the inherent edge structure of the underlying graphs. Nevertheless, there is a wide range of graph embedding methods available that spans from *spectral methods* [7, 8], over *graph kernels* [9, 10], to *dissimilarity embeddings* [11, 12], and *graph neural networks* [13, 14].

In the present paper, we propose a graph-based pattern recognition framework that directly operates in the graph domain without embedding. Our method is based on graph matching algorithms [1], and in particular, on *graph edit distance* (GED) [15, 16]. GED quantifies a graph dissimilarity on the basis of the minimum amount of modification required to transform a source graph into a target graph. Distance-based classifiers such as the *K-nearest neighbors classifier* (KNN) joined with GED have shown reasonable classification accuracies on diverse classification tasks (e.g., [17, 18]).

The goal of the present paper is to improve the classification performance of a KNN coupled with GED. To this end, we define a novel method that extracts extra information out of different reduced versions of the original graphs and combine this information in a multiple classifier system.

A preliminary version of the present paper appeared in [19]. We extend the paper in both method and experimental evaluation. In particular:

- We provide more comprehensive details and a formal definition of our novel framework.
- We use two rather than only one node centrality measure during the graph reduction process, viz. *PageRank* and *Betweenness*.
- We conduct extensive research to assess whether or not this additional selection criterion helps to achieve better classification accuracies.
- We combine reduced graphs from both subspaces (obtained with PageRank and Betweenness) as further ensemble strategy.
- We present and thoroughly discuss validation results.
- We evaluate the run time of our method and compare it to a reference system.

The remainder of the present paper is structured as follows. In “Graph matching”, we introduce the formal notion of graphs and briefly review graph matching techniques. In “Multiple classifier system based on reduced graphs”, we explain the details of our reduction method to produce differently reduced graphs and describe how the combination of the distances or obtained predictions is performed in the reduced graph subspaces. In “Experimental evaluation”, we present the setup of our experiments and discuss the main results of our experimental evaluation. In the last section, we conclude the paper and suggest possible extensions for future work.

## Graph Matching

In the present section, we formally define graph structures and also briefly review well-known graph matching methods (including the one actually employed in this paper).

### Graph Structure

A graph $G = (V, E)$ in a graph space ${\mathcal {G}}$ consists of a finite set of *n* nodes $V = \{v_1, \ldots , v_n \}$ and a set of edges $E \subset (V \times V)$ between these nodes. The size of a graph is usually defined as the cardinality of its node set $|V |= n$. If the graph is directed, an edge is defined as $(u, v) \in E$ with a starting node $u \in V$ and an end node $v \in V$. Otherwise, an edge is defined as $(u, v) \in E \leftrightarrow (v, u) \in E$ in the case of undirected graphs. A node (resp. edge) labeling function $\mu : V \mapsto L_V$ (resp. $\nu : E \mapsto L_E$) is defined in case of labeled nodes (resp. labeled edges). For example, the label alphabets $L_V$ and $L_E$ for both nodes and edges can be given by the set of integers $L = \{1, 2, 3, \ldots \}$, the vector space $L = {\mathbb {R}}^{n}$, or a set of symbolic labels $L = \{\alpha , \beta , \gamma , \ldots \}$.

In this paper, we focus our work on *simple, undirected graphs*, that is, graphs with at most one edge between pairs of nodes and no self-loops (i.e., edges between a node and itself). Note, however, that our method is in general applicable to any kind of graphs (i.e., directed, undirected, labeled, unlabeled, etc.)

### Graph Matching Methods

The present paper is concerned with structural pattern recognition with a strong focus on graph-based data representations [20]. The field of structural and graph-based pattern recognition has a long tradition [1, 21] and can roughly be subdivided into three areas, viz. *graph matching*, *graph kernel*, and *graph neural network*. The focus of our research is on the first area—graph matching.

*Graph matching* refers to the evaluation of the dissimilarity between two graphs. The overall aim of graph matching is to find a correspondence between the nodes and edges of two graphs that satisfies some, more or less, stringent constraints [1]. Standard procedures for testing graphs for *isomorphism*, for instance, are based on tree search techniques with backtracking [22, 23].

Tree search algorithms for (sub)graph isomorphism computation can also be adopted to so-called *error-tolerant graph matching* [24, 25]. Error-tolerant graph matching allows quantifying a subtle dissimilarity score between graphs even if they have no, or only very little, similarities in structure and labeling. *Graph edit distance*, is a prominent member of the family of error-tolerant approaches (details follow in the next subsection). Research on graph edit distance (and related measures) is still one of the most active fields in structural pattern recognition [18, 26, 27]. However, several other error-tolerant graph matching methods have been proposed in the literature. For instance, the authors of [28] introduce an error-tolerant graph matching that relies upon spectral features that encodes a graph as a bag of partial node coverages. In [29] a probabilistic graph matching method that is based on sequences of nodes of random walks is proposed. In [30] a new graph matching based on mutual information between graphs with a combination of copula functions is proposed. We refer to [1] for more extensive reviews on different graph matching methods.

#### Graph Edit Distance

In the graph-based framework proposed in the present paper, we employ *graph edit distance* (GED) [15, 16] as basic matching algorithm. Note, however, that our framework works independently of the actual graph matching algorithm. This means, in particular, that GED could be substituted with any other graph matching method available. In this subsection, we give in short an introduction and definition to GED (so that the paper remains self-explaining).

When comparing two graphs $G_1$ and $G_2$, GED computes the least amount of *edit operations* necessary to convert $G_1$ to $G_2$. In its original definition, three edit operations (namely *insertions*, *deletions*, and *substitutions*) are allowed on both nodes and edges. Employing those edit operations, GED computes an *edit path* $\lambda (G_1,G_2)$ between $G_1$ and $G_2$ as a set $\{e_1,\ldots , e_k\}$ of *k* edit operations $e_i$ that completely transform $G_1$ into $G_2$.

A cost function $c(\cdot )$ is commonly defined to weight the strength of each edit operation, and GED corresponds to the sum of costs of the edit path that minimizes the overall edit cost.

The problem of optimizing the overall cost of GED is known to be NP-complete for general graphs [31]. This means that the run time for finding the minimal cost edit path may be huge even for rather small graphs.

In recent years several *approximate*, or *suboptimal*, algorithms for error-tolerant graph matching have been proposed [31–36]. These algorithms offer polynomial, rather than exponential, run times. Yet, in contrast to *optimal* error-tolerant graph matching, suboptimal algorithms do not guarantee to find the global minimum of the matching cost, but only a local one.

Another common way to make error-tolerant graph matching more efficient is to restrict considerations to special classes of graphs. Examples include the classes of ordered graphs [37], planar graphs [38], or trees [39].

In the present paper, we use the suboptimal algorithm BP for GED computation which is applicable to virtually all types of graphs [34, 40, 41]. BP is based on an (optimal) fast optimization procedure mapping nodes and their local structure of one graph to nodes and their local structure of another graph. The algorithm BP offers cubic time complexity and is a widely used method in the field of graph based pattern recognition [42].

## Multiple Classifier System Based on Reduced Graphs

**Fig. 1:**
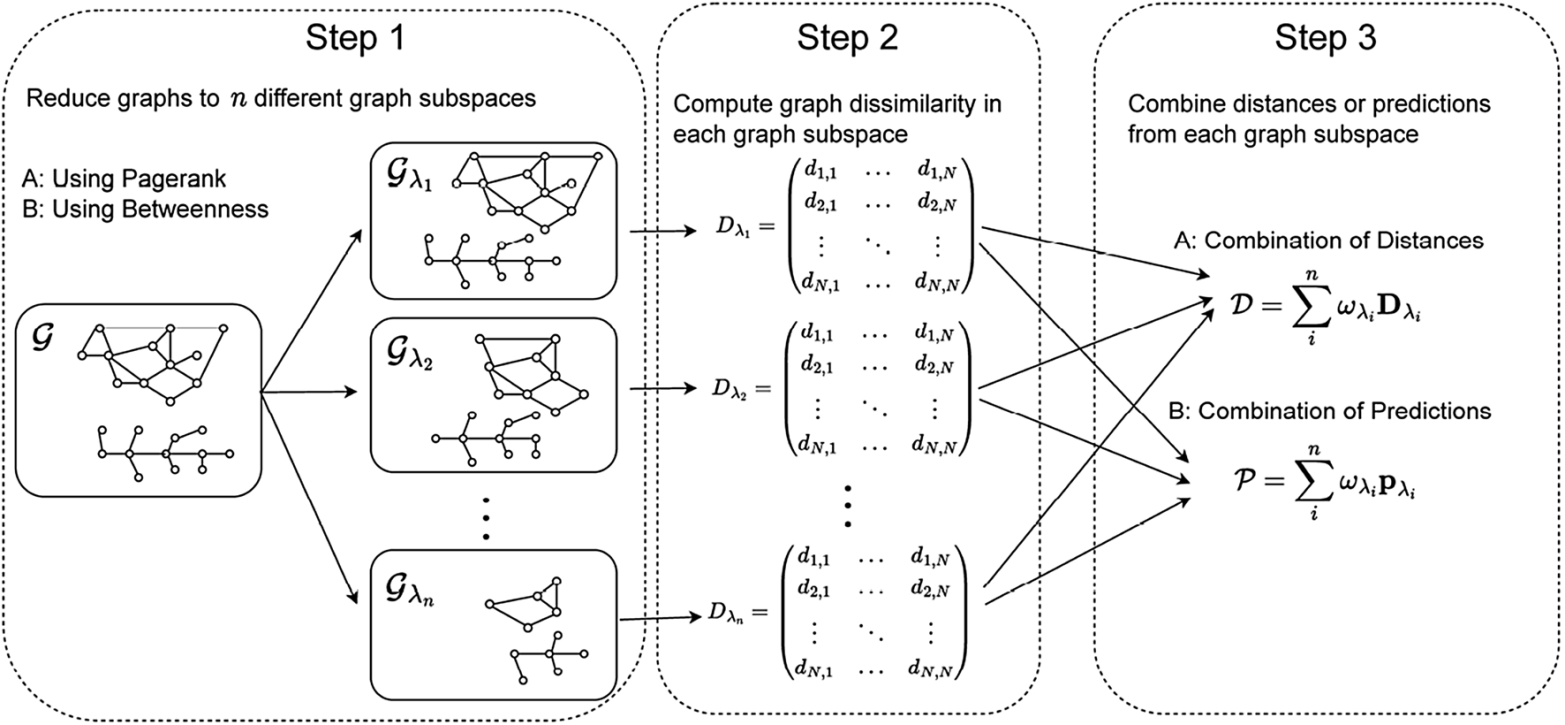
Three basic steps of our novel framework: (1) graph reduction to obtain the reduced graph subspaces, (2) graph matching in reduced graph subspaces and (3) building a multiple classifier system

In the present section, we thoroughly introduce and describe our novel framework. Roughly speaking, our method is based on three basic steps (as illustrated in Fig. 1).

- First, we create various reduced graph subspaces, that contain graphs that are in turn reduced to the nodes that contribute the most to the original graph structure. We employ two node centrality measures, viz. PageRank and Betweenness, as node selector criterion during the reduction process.
- The second step consists of computing a graph dissimilarity between the graphs in the reduced graph subspaces. For this purpose, we use the concept of GED as covered in “Graph Edit Distance”.
- The third and last step of our procedure consists in linearly combining either the distances or the predictions obtained in the different graph subspaces. Any classification method that makes use of GED in some way can be used for this purpose (e.g., distance based graph kernels or distance based classifiers such as the K-nearest neighbor classifier).

From a broad perspective, the proposed framework as shown in Fig. 1 is somehow related to the recently introduced hierarchical graph matching framework [43].^1^ In this framework, the nodes are aggregated into super nodes during a graph compression process. A hierarchy of compressed graphs is constructed by means of a community detection algorithm. Then, the matching is performed, starting at the most compressed graphs and potentially going up level by level if a certain similarity threshold is exceeded. In our method, however, we make use of reduced graphs, i.e., nodes are omitted/deleted during the graph reduction procedure (rather than combined via compression). To this end, we quantify the structural information of each node via centrality measures adapted from network technology (formally introduced in the next section). Next, we remove the nodes that contribute the least to the structure of the graph (according to the centrality measure actually applied). Moreover, we use the extra information gained from the reduced graph subspaces in a multiple classifier scenario and do not perform a coarse-to-fine classification.

Our method of graph reduction might result in isolated nodes and/or graphs that are divided into several connected components. Yet, as we use GED for matching the reduced graphs, this is not an obstacle (as GED can handle both isolated nodes and graphs that are not connected).

In the following three subsections (“Graph reduction, Graph matching in reduced graph spaces, and Building a multiple classifier system”), the three basic steps of our framework are described in greater detail.

### Graph Reduction

Our approach crucially relies upon reduced graph subspaces. We define a particular graph subspace as the set of graphs reduced to a given percentage of their original size. Hence, we need a fast yet deterministic way of creating graphs of reduced sizes. The proposed reduction method is based on network’s node *centrality measures* [5].

Centrality measures indicate how important a node in a graph is by quantifying the contribution of each node to the graph connection. Roughly speaking there are two categories of centrality measures available, viz. *Degree-based* and *Shortest-path based* measures. The degree-based methods use the degree property of the nodes, i.e., how many edges are connected to a node, to derive their centrality score. Meanwhile, the shortest path based algorithms compute a node’s centrality score by counting the number of paths between any two pairs of nodes that passes by it. In the present paper, we focus on two popular centrality measures stemming from both categories, namely *PageRank* (degree-based) [46] and *Betweenness* (shortest path based) [47]. Note that our graph reduction framework is not only limited to those two measures. That is, any other centrality measures could be used as well.

The basic idea behind PageRank is that the importance of a node increases by being connected to other nodes that are themselves important. The importance of a node is thus proportional to the sum of the scores of the nodes in its neighborhood. A problem of this definition is, however, that if an influential node is linked to many other nodes then its high-centrality will be widespread among all its neighbors. To counter this issue the authors of [46] propose to dilute the influence of an influential node proportionally to the number of its neighbors. Formally, the vector ${\varvec{x}}$ that contains the *n* PageRank scores for all nodes $\{v_1, \ldots , v_n \}$ of a given graph $G = (V, E)$ is defined by

1 $$\begin{aligned} {\varvec{x}} = \alpha {\varvec{A}} {\varvec{D}}^{-1} {\varvec{x}} + \beta {\varvec{1}} , \end{aligned}$$

where

- $\alpha$ is used as a damping factor (there is no clearly defined theory to choose its value, it is often set to 0.85 as proposed in [46]).
- ${\varvec{A}}$ corresponds to the adjacency matrix of the graph *G*.
- ${\varvec{D}}$ is the diagonal matrix with elements $D_{ii} = \max (d_{i}^\textrm{out}, 1)$, where $d_{i}^\textrm{out}$ corresponds to the outdegree of the *i*th node.
- $\beta$ is an additive constant (we conventionally set it to 1).

The betweenness centrality measure counts how many times a node lies on the shortest paths connecting pairs of nodes. In a graph with flowing information, it indicates, on average, the number of time messages passes between each pair of nodes. Formally, the betweenness score $x_i$ for the *i*th node $v_i \in V$ of a given graph $G = (V, E)$ is defined by

2 $$\begin{aligned} x_i = \sum _\textrm{st} n_\textrm{st}^{i}, \end{aligned}$$

where $$n_\textrm{st}^{i} = {\left\{ \begin{array}{ll} 1, &{} \quad \text {if node }v_i\text { lies on the shortest path between node }v_s \in V\text { and }v_t \in V \\ 0, &{} \text {otherwise} \end{array}\right. }$$

Once the node centrality scores are computed for each node (either with PageRank or with Betweenness), we sort them according to their centrality from the least to the most important one. With a reduction factor $\lambda \in [0, 1]$ we are then readily able to select the most influential $\lfloor \lambda |V |\rfloor$ nodes in the graph, while the other nodes and their incident edges can be removed from the graph.

The reduction factor $\lambda$ can be seen as the percentage of remaining nodes of the original graph. That is, if we set $\lambda =0.8$, then around $80\%$ of the nodes remain in the reduced versions of the graphs. We can now arbitrarily vary the reduction factor $\lambda$ from 1.0 to 0.0 by different step-sizes (e.g., $\lambda \in \{1.0, 0.8, 0.6, 0.4, 0.2\}$) to obtain differently sized graphs out of one source graph. Note that with $\lambda = 1.0$ all the nodes remain in the reduced graph which obviously corresponds to the original graph.

**Fig. 2:**
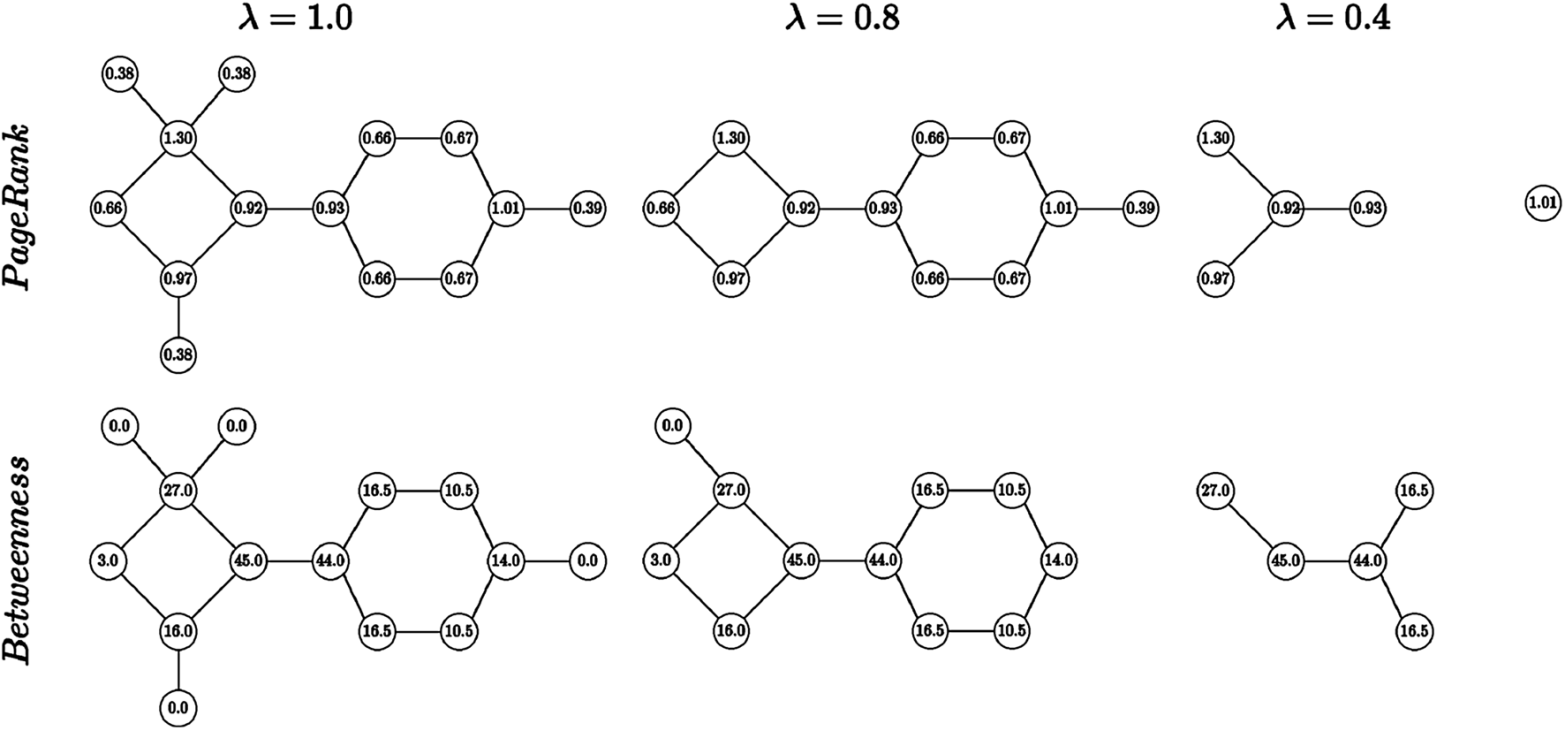
Illustrative example of our reduction scheme on an artificial graph with both node selection criteria (i.e., PageRank and Betweenness) and $\lambda \in \{0.8, 0.4\}$. (For better visibility, the PageRank scores are scaled up by factor 10)

In an illustrative example displayed in Fig. 2, we show an original graph $g = (V, E)$ with $|V |= 14$ and two reduced versions of the graph with $\lambda = 0.8$ and $\lambda = 0.4$. The number of nodes that remain in the graph with $\lambda = 0.8$ and $\lambda = 0.4$ is $\lfloor 0.8 \cdot 14 \rfloor = 11$ and $\lfloor 0.4 \cdot 14 \rfloor = 5$, respectively.

In the remainder of this paper, we term a reduced graph (with reduction factor $\lambda$) as $G_{\lambda } = (V_{\lambda }, E_{\lambda })$. When reducing all graphs in a given data set of size *N*, we obtain a reduced graph subspace ${\mathcal {G}}_{\lambda } = \{G_{\lambda }^{(1)}, \ldots ,G_{\lambda }^{(N)}\}$. Repeating this process with different reduction factors $\lambda _1, \ldots , \lambda _n$ we obtain *n* reduced graph subspaces ${\mathcal {G}}_{\lambda _1}, \ldots ,{\mathcal {G}}_{\lambda _n}$.

### Graph Matching in Reduced Graph Spaces

Given the *n* different graph subspaces ${\mathcal {G}}_{\lambda _1}, \ldots ,{\mathcal {G}}_{\lambda _n}$, we can now compute pairwise graph dissimilarities in each graph subspace ${\mathcal {G}}_{\lambda _i}$. To this end, we employ the GED approximation BP as outlined in “Graph matching”. In detail, for each graph $G^{(j)}_{\lambda _i} \in {\mathcal {G}}_{\lambda _i}$ we create a distance vector $d_j = [d_{j, 1}, d_{j, 2},\ldots , d_{j, N}]$ representing the distances between itself and the *N* other graphs in ${\mathcal {G}}_{\lambda _i}$ and merge the obtained vectors to produce a distance matrix ${\varvec{D}}_{\lambda _{i}}$.

### Building a Multiple Classifier System

Once the distance matrices ${\varvec{D}}_{\lambda _{i}}$ for each reduction level $\lambda _{i}$ are obtained, we employ two different procedures to combining them and getting a final classification result. In both of the combination scenarios, we employ a distance-based classifier, viz. a *K*-nearest-neighbor classifier (KNN). The KNN is clearly advantageous in our framework because it directly operates on the resulting distances and can also be used as an indicator of the underlying quality of the distances. Both combination procedures are described in detail in the following paragraphs.

The first combination procedure consists of linearly combining the multiple distance matrices ${\varvec{D}}_{\lambda _{1}}, \ldots , {\varvec{D}}_{\lambda _{n}}$ at different levels $\lambda _{1}, \ldots , \lambda _{n}$ into one *meta-distance matrix* defined by

3 $$\begin{aligned} {\mathcal {D}} = \sum ^{n}_{i} \omega _{\lambda _{i}} {{\textbf {D}}}_{\lambda _{i}}, \end{aligned}$$

where the parameter $\omega _{\lambda _{i}} \in [0, 1]$ weights the influence of each reduced graph subspace ${\mathcal {G}}_{\lambda _{i}}$. Matrix ${\mathcal {D}}$ is eventually fed into a KNN to perform the final classification.

The second idea for condensing the *n* different graph subspaces ${\mathcal {G}}_{\lambda _{1}}, \ldots , {\mathcal {G}}_{\lambda _{n}}$ consists of combining the predictions obtained from the KNN at each reduced graph subspace.

Formally, we obtain a prediction vector ${\varvec{p}}_{\lambda _{i}} = [p_1, p_2,\ldots , p_N]^{T}$ for each graph subspace ${\mathcal {G}}_{\lambda _{i}}$ where $p_j$ with $j = 1, \ldots , N$ corresponds to the prediction of the *j*th graph in the graph subspace ${\mathcal {G}}_{\lambda _{i}}$. The prediction vectors are finally linearly combined by

4 $$\begin{aligned} {\mathcal {P}} = \sum ^{n}_{i} \omega _{\lambda _{i}} {{\textbf {p}}}_{\lambda _{i}} \end{aligned}$$

to obtain the final classification result. That is, we conduct a weighted majority voting as proposed in [48].

To weight the influence of each reduced graph subspace ${\mathcal {G}}_{\lambda _{i}}$ both combination methods introduced above make use of a vector ${\varvec{\omega }} = (\omega _{\lambda _1}, \dots , \omega _{\lambda _n})$, that incorporates the *n* weighting factors $\omega _{\lambda _i}$ for all graph subspaces.

Our goal is to linearly combine the *n* reduced graph subspaces, and thus we apply further constraints on ${\varvec{\omega }}$ such that each entry $\omega _{\lambda _i} \in {\varvec{\omega }}$ is comprised in a range between 0 and 1 and the sum of all weights equals 1. Formally

5 $$\begin{aligned} \begin{aligned}&\sum ^{n}_{i=1} \omega _{\lambda _i} = 1 \\&\quad \text { and } \\&\omega _{\lambda _i} \in ]0, 1[&\quad \forall i = 1, \ldots , n \end{aligned} \end{aligned}$$

We aim to find the linear coefficient vector ${\varvec{\omega }}^{*}$ such that the combined distance matrix ${\mathcal {D}}$ or the combined predictions ${\mathcal {P}}$ lead to the best possible classification accuracy. We use two different optimization strategies to find ${\varvec{\omega }}^{*}$.

The first optimization method consists of a search over the parameter space in grid-search fashion. Unfortunately, grid-search is not an efficient technique and scales poorly when the search space is large. In our specific case the search space has a size of ${\mathcal {O}}(\textrm{D}^{n})$, where $\textrm{D}$ is the total number of values that a weight $\omega _{\lambda _i} \in {\varvec{\omega }}$ can take and *n* is the number of graph subspaces that are potentially combined.

As a second optimization technique we use a *genetic algorithm* (GA) [49]. GAs are more efficient and scalable search procedures over large search spaces than grid search approaches. Therefore, by means of GAs, we are able to explore more subtle combinations of the weights and thus potentially obtain better classification accuracies. Yet, the optimality of the found solution is not guaranteed. It also has the disadvantage to suffer from overfitting and there is no well-defined regularization procedure to prevent it. In this scenario, we define ${\varvec{\omega }}$ as the so-called *chromosome* where each entry $\omega _{\lambda _i} \in {\varvec{\omega }}$ represents a *gene*. We set the *fitness function* of a chromosome to be the classification accuracy of the KNN and allow both operations *mutations* and *cross-overs*.

## Experimental Evaluation

### Data Sets

During the evaluation phase of our novel procedure, we use six data sets from different domains. The AIDS and Mutagenicity data sets are retrieved from the IAM graph database [50]^2^ and the four other data sets (i.e., NCI1, Proteins, Enzymes, IMDB Binary) are retrieved from the TUDataset graph repository [51]^3^ In Table 1, we show some statistical properties for each graph data sets (the number of graphs, the number of graphs per split used for training, validation and testing, the number of classes, and the average number of nodes and edges)

The first three data sets (AIDS, mutagenicity, and NCI1) represent molecules stemming from two classes. The two categories from the AIDS data set represent molecules that may have an effect against the HI virus or not. The graphs from the mutagenicity data set represent molecules that may have mutation properties, and the graphs in the NCI1 data set represent molecules that are able (or not) to diminish the expansion of tumorous cells. The graphs from the Proteins data set correspond to proteins that are classified as enzymes or non-enzymes. The Enzymes graphs represent tertiary proteins stemming from six enzyme classes [3] The IMDB-binary graphs encode social networks representing movie collaborations between different actors/actresses.

**Table 1:** Properties of the graph data sets

Data set	$|G |$ (tr, va, te)	$|\Omega |$	$\emptyset |V |$	$\emptyset |E |$
AIDS	2000 (250, 250, 1500)	2	9.5	10.0
Mutagenicity	4337 (1,500, 500, 2337)	2	30.3	30.8
NCI1	4110 (1500, 500, 2110)	2	29.9	32.3
Proteins	1113 (660, 220, 223)	2	39.1	72.8
Enzymes	600 (360, 120, 120)	6	32.6	62.1
IMDB binary	1000 (600, 200, 200)	2	19.8	96.5

We show the number of graphs ($|G |$) with the size of the training, validation and test set (tr, va, te), the number of classes ($|\Omega |$) and the average number of nodes and edges ($\emptyset |V |$, $\emptyset |E |$) per data set

We split all data sets into three disjoint subsets for training, validation, and testing as follows. We split the graphs from the IAM graph repository according to the proposed splitting of the benchmark. The NCI1 data set is split to match the size of the three sets of the Mutagenicity data set. The other data sets are divided with respect to the 60–20–20% split rule for training, validation, and test sets, respectively.

### Experimental Setup and Validation Process

The main purpose of the following experiments is to empirically verify whether or not the information extracted out of the reduced graphs can help to improve the overall classification performance. To test this hypothesis, we first build a baseline for our evaluation by running a KNN classifier on the original graphs.

The individual hyperparameters of the KNN are optimized with the graphs contained in the validation set. To alleviate overfitting during the optimization process, we apply a fivefold cross-validation. The parameters to optimize consist of $\alpha \in ]0, 1[$ that weights the relative influence of node and edge edit operation costs and $k \in \{1, 3, 5\}$ that corresponds to the number of neighbors used by the KNN. We show the optimal parameters $\alpha$ and *k* found for each data set in Table 2.

**Table 2:** Optimal values for $\alpha$ and *k* obtained during the hyperparameter optimization on the validation sets

Data set	${\alpha }$	*k*
AIDS	0.7	1
Mutagenicity	0.6	5
NCI1	0.7	5
Proteins	0.9	3
Enzymes	0.9	1
IMDB binary	0.9	5

For our novel framework, we use the optimized hyperparameters $\alpha$ and *k* computed during the optimization phase on all graph subspaces ${\mathcal {G}}_{\lambda _1}, \ldots ,{\mathcal {G}}_{\lambda _n}$. We set the reduction factors to $\lambda \in \{1.0, 0.8, 0.6, 0.4, 0.2\}$ to obtain the original graph space and four reduced graph subspaces with both PageRank and Betweenness. In our evaluation the five graph (sub)spaces, reduced with PageRank and Betweenness, are either used individually or combined with each other. In the combined case, we obtain a total of nine graph (sub)spaces (the original graph space and four subspaces per centrality measure).

When optimizing the weighting parameters ${\varvec{\omega }}$ with grid search we use the five reduction levels presented above in conjunction with 11 possible weighting factors, i.e. $\omega _{\lambda _i} \in \{1.0, 0.9, \ldots , 0.1, 0.0\}$. Only with those reduction factors the search space is already quite large (having $11^5 = 161,051$ different possibilities). Because of the exponential growth of the search space we cannot apply the grid-search procedure in the scenario where we combine PageRank and Betweenness graph subspaces (in this case the search space would have a size of $11^9 \approx 2.9$ billion possibilities which is no longer feasible).

For the GA optimization, we use a random initial population of 30 individual chromosomes, where the random weights of each chromosome (i.e., the genes) are defined such that they sum up to one to match the weighting constraints. Furthermore, the crossover sites in each iteration of the GA are randomly chosen, the mutation probability $p_m$ is set to 0.1, and we run the GA for 100 iterations.

In Table 3 we summarize all the reduction, combination, and optimization methods discussed above. We are now able to combine all the presented methods with each other. For instance, we can create a system termed *PR-CoD-GS* that associates PageRank with the combination of distances and a grid search optimization. As stated above, the grid search optimization is not applicable to the combined reduction, leading to a total of ten different experimental setups.

**Table 3:** Different reduction, combination, and optimization methods to create ten experimental setups

Reduction methods	Combination methods	Optimization methods
PageRank (PR)	Combination of distances (CoD)	Grid search (GS)
Betweenness (BW)	Combination of predictions (CoP)	Genetic algorithm (GA)
PageRank + betweenness (PR + BW)$^{\textrm{a}}$		

$^{\textrm{a}}$Due to computational reasons this combined reduction is only optimized via GA

**Fig. 3:**
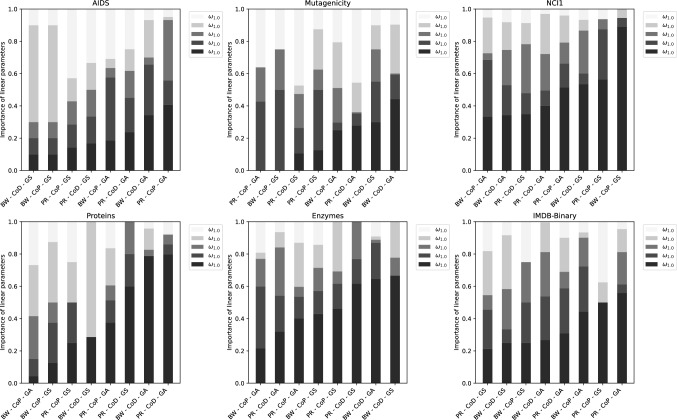
Importance of the individual graph subspaces in the linear combination for all data sets as well as all reduction, combination and optimization methods

In Fig. 3, we show a bar plot that displays the individual weights ${\varvec{\omega }}^{*}$ obtained after the optimization procedure. The figure exhibits the influence of the individually reduced subspaces for all conducted experiments.

In some cases, we observe that the original graph subspace ${\mathcal {G}}_{\lambda _{1.0}}$ dominates the other subspaces. This trend is particularly apparent, for instance, on the Enzymes and NCI1 data sets with *BW-CoD-GS* and *BW-CoP-GS*, respectively. On the other hand, we can observe in some cases that the reduced graph subspaces substantially contribute to the combined distances and/or predictions. For instance, on the Mutagenicity or Proteins data sets with *BW-CoP-GS*, the original graph space is completely omitted or does not substantially influence the final classification.

Yet, in tendency, no clear pattern in the weighting factors is visible that could favor any of the graph subspaces. Thus the optimal weighting parameters have to be found in an empirical fashion. This observation may indicate that all the reduced subspaces are somehow important and that the optimal weighting depends on the actual application.^4^

### Qualitative Analysis of the Reduced Graphs

**Fig. 4:**
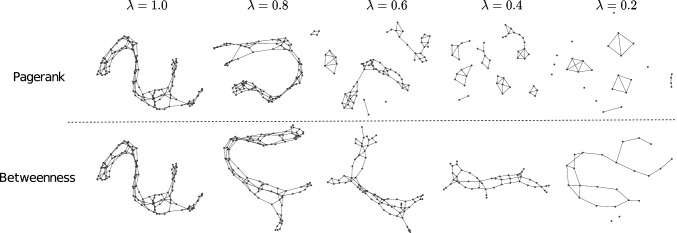
Reduced molecular compound from the enzyme data set

**Fig. 5:**
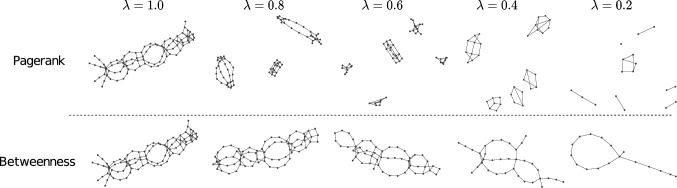
Reduced molecular compound from the proteins data set

In Figs. 4 and 5, we show examples of graph reductions with PageRank and Betweenness at different reduction levels $\lambda$ on two data sets.^5^

We observe noticeable differences between the two centrality measures. When reducing the graphs based on the PageRank selection method, we generally preserve the intra-community nodes. That is, communities are kept together while being separated from each other. Roughly speaking, PageRank tends to produce reduced graphs with large numbers of connected components. This effect is particularly apparent in Fig. 4.

On the other hand, by deleting nodes upon low betweenness values, we keep the backbone structure of the graph. That is, we observe that the main paths in the graph form communities and are kept when discarding nodes from the graph. Simultaneously, the external nodes from the communities are omitted. This effect is particularly visible in Fig. 5.

### Results on the Test Sets

In Table 4, we present the classification accuracies obtained on all test sets by our method that combines either the distances, termed *combination of distance matrices (CoD)*, or the predictions, termed *combination of predictions (CoP)*. Both combinations are either applied on PageRank (PR), betweenness (BW), or PageRank and betweenness (PR + BW) reduced graphs. Additionally, we present individual results for both optimization strategies, viz. grid search (GS) and genetic algorithm (GA) (note that for PR + BW only the GA optimization is applied due to computational reasons).

**Table 4:** Classification accuracy [%] obtained on the test set with linear combinations of reduced graphs

Data set	AIDS	Mutagenicity	NCI1	Proteins	Enzymes	IMDB-binary
Method						
Baseline KNN	98.53	71.33	70.33	73.82	41.67	66.00
PR $-$ CoD $-$ GS	99.13$\circ$	71.84	72.09	73.39	45.83	64,50
PR $-$ CoD $-$ GA	99.13${\circ }$	**72**.**66**	**73**.**22**$\circ$	75.54	48.33$\circ$	66.00
PR $-$ CoP $-$ GS	**99**.**33**$\circ$	72.32	70.52	73.82	41.67	**70**.**00**
PR $-$ CoP $-$ GA	99.13$\circ$	71.84	70.52	76.39$\circ$	37.50$\cdot$	**70**.**00**
BW $-$ CoD $-$ GS	98.07	71.25	71.28	69.52	46.67	64.00
BW $-$ CoD $-$ GA	99.20$\circ$	72.53	71.56	75.53	**49**.**18**$\circ$	64.00
BW $-$ CoP $-$ GS	98.07	71.29	70.52	76.82$\circ$	43.33	65.50
BW $-$ CoP $-$ GA	99.20$\circ$	71.59	70.24	73.82	41.67	65.50
PR + BW $-$ CoD $-$ GA	99.13$\circ$	**72**.**66**	71.89	75.54	48.33$\circ$	65.50
PR + BW $-$ CoP $-$ GA	99.27$\circ$	71.72	69.38	**77**.**25**$\circ$	40.83	65.00

We present the results obtained by a KNN for the baseline and our two combination methods that are combination of distances (CoD) and combination of predictions (CoP). The best result per data set is shown in boldface. ($\circ$/$\cdot$: statistically significantly better/worse than the baseline on a 5% level using a *Z*-test)

We start our discussion with a focus on the Pagerank reduced graphs. We observe that at least one of the proposed combinations of distances or predictions of the reduced subspaces improves the classification accuracy compared to the baseline on all data sets. In 21 out of 24 comparisons our novel approach achieves better results than the reference system (6 of these improvements are statistically significant). These significant improvements are observed on four different data sets. On the other hand we observe only three deteriorations of which only one is significant. Last but not least, we observe that on four data sets the combination of PageRank reduced graphs achieves the overall best results (shown in bold face). Two of these overall best results are achieved with distance based and two with prediction based combinations.

In the case of combinations of betweenness reduced graphs, comparable, yet slightly worse, results as with the PageRank reduction are obtained. That is, with Betweenness we observe only in 13 out ouf 24 comparisons an improvement over the reference system. On the Enzymes data set, however, the classification accuracy is substantially improved by about 8 percentage points when compared to the baseline (from 41.67% to 49.18%).

When combining both PageRank and betweenness graph subspaces, we observe eight improvements in total when compared to the baseline. Three of these improvements are statistically significant. Moreover, with this particular combination we obtain overall best results on the Mutagenicity and Proteins data sets.

To assess which reduction method (PR or BW) together with which combination method (CoD or CoP), coupled with which optimization procedure (GS or GA) performs the best, we rank all methods per data set and sum up the ranks per method. We can report two clear winners that achieve the smallest sum of rank points, viz. *PR-CoD-GA* and *PR+BW-CoD-GA*. It is remarkable that PageRank plays at least a role in both winners and that both winners are based on the distance combination that is optimized via genetic algorithm. At the opposite end of the ranking, we have *BW-CoD-GS* and *BW-CoP-GA*.

Actually, if we consider each individual graph subspace as an individual system, we obtain an ensemble of classifiers. It is well-known that ensemble learners are more robust to potential noise and offer better generalization power than individual classifiers. As the proposed method exploits the power of ensemble learners, this gives us a possible and plausible explanation of the substantial improvement of the novel method compared to the reference system.

In summary, we can report that the GA optimization method achieves better results than the grid search, the combination of distances performs better than the combination of predictions, and PageRank works better than Betweenness for building the reduced graph subspaces.

### Time Analysis

The main drawback of our novel three-step method is the extra computation time used to compute GED in the different graph subspaces. The computation of GED is actually the bottleneck of our framework in terms of time complexity (although using an ${\mathcal {O}}(n^3)$ approximation algorithm where $n = |V |$). Hence, we focus our runtime analysis on the second step of our framework.

**Fig. 6:**
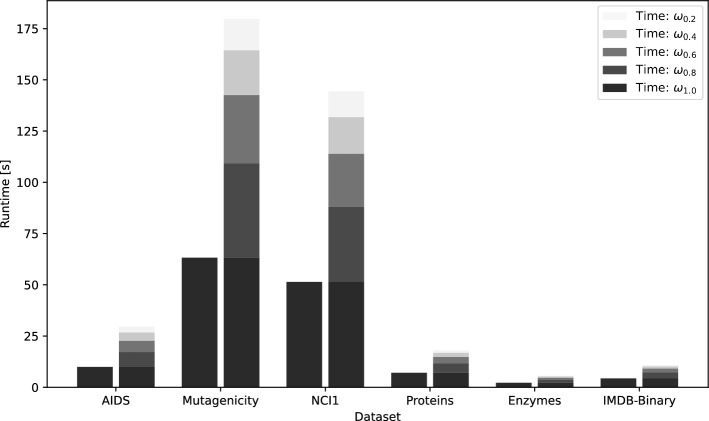
Runtime of GED computation for each graph subspace per data set

Based on the fact that the reduced graphs have by definition fewer nodes, the run time of GED is supposed to decrease the smaller the graph subspaces are. In Fig. 6, we show the runtime of GED for each graph subspace per data set. We observe that in 4 out of 6 data sets (i.e., AIDS, proteins, enzymes, and IMDB-Binary) the execution time is only about twice slower compared to the runtime of GED computation on the original graphs. On the other two data sets (Mutagenicity and NCI1) the run time is about three times slower than the original system. Considering that our combined systems are superior to the reference system, one can certainly argue that the higher runtime is worth it in any case.

## Conclusion and Future Work

In the present paper, we propose a novel framework for graph-based pattern recognition that combines extra information gained from reduced graph subspaces. Roughly speaking, the proposed method works in three subsequent steps. In the first step, we produce multiple reduced graph subspaces using graph reduction methods. The graph reduction consists of first evaluating the node importances by means of PageRank or Betweenness and then removing the least important ones. During the second step, we use GED to compute the distances between the graphs in their corresponding reduced graph subspaces. In the last step, we linearly combine either the distances or the predictions obtained in the differently reduced graph subspaces. The linear coefficients for the combination are either optimized by means of a grid search or a genetic algorithm.

We empirically validate the advantage of our novel method by performing an evaluation on data sets from a broad range of domains. In particular, we show that a KNN classifier clearly benefits from the combination of the distances or predictions of reduced graphs. That is, on all data sets the proposed algorithmic framework outperforms the reference system by several percentage points. Comparing the different subsystems with each other, we conclude that the PageRank reduction in conjunction with the combination of distances optimized via genetic algorithm is a good choice in general.

Regarding the importance of each graph subspace we can conclude that all of them are somehow important. The actual importance seems to depend on both the data set and optimization process.

Clearly, the increase in computation time is the major drawback of the proposed system. The runtime of our novel framework is actually higher than that of the reference system, but not five times higher, as one might have expected at first glance. The reason for this is, of course, the dramatic decrease of the runtime in strongly reduced graph subspaces. Overall, we observe runtimes that are twice or at most three times as high as those of the reference system. Considering the significantly improved classification accuracy, this slowdown seems acceptable.

In future work, we plan to employ other reduction processes in our framework. In particular, methods which might produce reduced graphs that contain more relevant information within its reduced structure. A learning-based reduction method would be a good candidate, for instance. Such a method might learn which node to delete to conserve as much information as possible in the structure of the reduced graph. A learning-based method would possibly be also advantageous regarding the runtime. Once trained, such a system would allow our framework to scale to very large graphs.

A complementary idea is to embed graphs (implicitly or explicitly) into a vector space and then carry out the combination of distances (e.g., by means of a kernel machine). For instance, each (reduced) graph can be embedded into a vector space using a specific kernel. Once the (reduced) graphs are embedded, one could instantly apply the second and third step our framework in the embedding space.

Another line of research includes to further investigate the runtime of our framework when working with large graphs (e.g., $|V |> 1000$). It would be particularly interesting to measure the extra-time required for the computation of GED in the individual graph subspaces. This evaluation could give us insight on a general trend of the runtime by showing the computational issues of GED on large graphs and revealing the substantial reduction of the runtime in the reduced graph subspaces.

## Figures and Tables

**Fig. 7:**
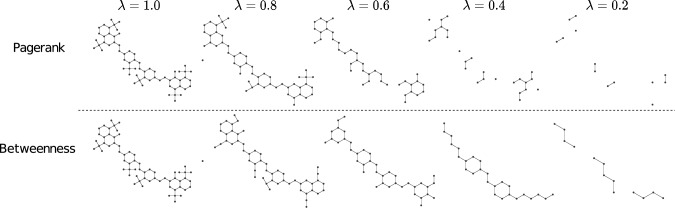
Reduced molecular compound from the AIDS data set

**Fig. 8:**
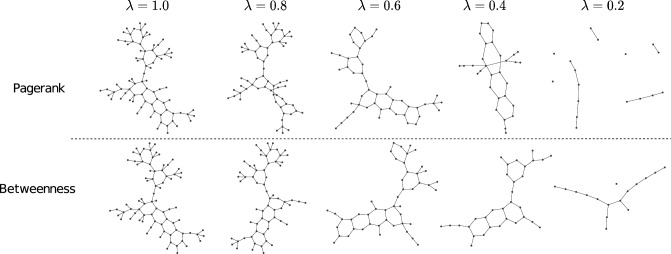
Reduced molecular compound from the mutagenicity data set

**Fig. 9:**
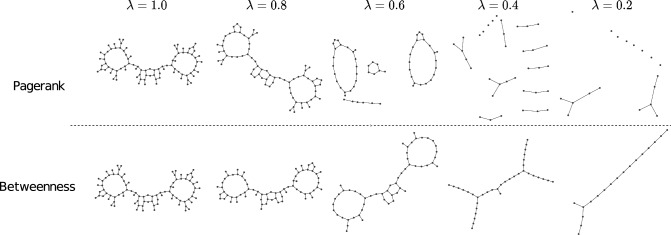
Reduced molecular compound from the NCI1 data set

**Fig. 10:**
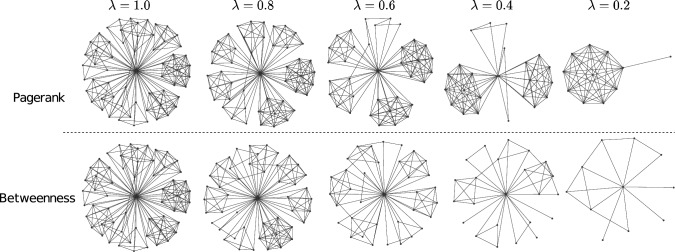
Reduced network from the IMDB data set

## Data Availability

All the datasets used in the experimental evaluation of this paper are freely available.

## Appendix A: Examples of Reduced Graph

See Figs. 7, 8, 9 and 10.

## Footnotes

1 Other hierarchical graph representations are presented in [44, 45], for instance.

2 www.iam.unibe.ch/fki/databases/iam-graph-database.

3 http://www.graphlearning.io/.

4 We also study the weighting factors of each graph subspace when combining PageRank and Betweenness graph subspaces. Yet, no clear trend appears in the visualization and thus we do not display those results here.

5 On the other data sets similar observations can be made (see Appendix A).

## References

[1] Foggia P, Percannella G, Vento M (2014). Graph matching and learning in pattern recognition in the last 10 years. Int J Pattern Recognit Artif Intell..

[2] Gaüzère B, Brun L, Villemin D (2012). Two new graphs kernels in chemoinformatics. Pattern Recognit Lett..

[3] Borgwardt KM, Ong CS, Schönauer S, Vishwanathan SVN, Smola AJ, Kriegel H. Protein function prediction via graph kernels. In: Proceedings 13th international conference on intelligent systems for molecular biology 2005, Detroit, 25–29 June 2005. 2005. p. 47–56. 10.1093/bioinformatics/bti1007. 10.1093/bioinformatics/bti1007 15961493

[4] Kostakis O (2014). Classy: fast clustering streams of call-graphs. Data Min Knowl Discov.

[5] Newman MEJ (2010). Networks: an introduction.

[6] Vento M (2015). A long trip in the charming world of graphs for pattern recognition. Pattern Recognit.

[7] Yang C, Feng Y, Li P, Shi Y, Han J. Meta-graph based HIN spectral embedding: Methods, analyses, and insights. In: IEEE international conference on data mining, ICDM 2018, Singapore, November 17–20, 2018. 2018. p. 657–66. 10.1109/ICDM.2018.00081.

[8] Qiu H, Hancock ER (2006). Graph matching and clustering using spectral partitions. Pattern Recognit..

[9] Kriege NM, Johansson FD, Morris C (2020). A survey on graph kernels. Appl Netw Sci..

[10] Nikolentzos G, Siglidis G, Vazirgiannis M (2021). Graph kernels: a survey. J Artif Intell Res.

[11] Riesen K, Bunke H (2010). Graph classification and clustering based on vector space embedding. Series in machine perception and artificial intelligence.

[12] Livi L, Rizzi A, Sadeghian A (2014). Optimized dissimilarity space embedding for labeled graphs. Inf Sci.

[13] Wu Z, Pan S, Chen F, Long G, Zhang C, Yu PS (2021). A comprehensive survey on graph neural networks. IEEE Trans Neural Netw Learn Syst.

[14] Zhou J, Cui G, Hu S, Zhang Z, Yang C, Liu Z, Wang L, Li C, Sun M (2020). Graph neural networks: a review of methods and applications. AI Open.

[15] Bunke H, Allermann G (1983). Inexact graph matching for structural pattern recognition. Pattern Recognit Lett..

[16] Sanfeliu A, Fu K (1983). A distance measure between attributed relational graphs for pattern recognition. IEEE Trans Syst Man Cybern.

[17] Maergner P, Pondenkandath V, Alberti M, Liwicki M, Riesen K, Ingold R, Fischer A, Bai X, Hancock ER, Ho TK, Wilson RC, Biggio B, Robles-Kelly A (2018). Offline signature verification by combining graph edit distance and triplet networks. Structural, syntactic, and statistical pattern recognition—joint IAPR international workshop, S+SSPR 2018, Beijing, China, August 17–19, 2018, proceedings. Lecture notes in computer science.

[18] Fuchs M, Riesen K. Matching of matching-graphs—a novel approach for graph classification. In: 25th international conference on pattern recognition, ICPR 2020, virtual event, Milan, January 10–15, 2021, IEEE; 2020. p. 6570–76. 10.1109/ICPR48806.2021.9411926.

[19] Gillioz A, Riesen K, Marsico MD, di Baja GS, Fred ALN (2022). Improving graph classification by means of linear combinations of reduced graphs. Proceedings of the 11th international conference on pattern recognition applications and methods, ICPRAM 2022, online streaming, February 3–5, 2022.

[20] Riesen K, Fischer A, Bunke H, Schwenker F, Abbas HM, Gayar NE, Trentin E (2016). Approximation of graph edit distance by means of a utility matrix. Artificial neural networks in pattern recognition—7th IAPR TC3 workshop, ANNPR 2016, Ulm, Germany, September 28–30, 2016, proceedings. Lecture notes in computer science.

[21] Brun L, Foggia P, Vento M (2020). Trends in graph-based representations for pattern recognition. Pattern Recognit Lett..

[22] Carletti V, Foggia P, Percannella G, Ritrovato P, Vento M (2021). Two parallel versions of VF3: performance analysis on a wide database of graphs. Pattern Recognit Lett..

[23] Carletti V, Foggia P, Saggese A, Vento M (2018). Challenging the time complexity of exact subgraph isomorphism for huge and dense graphs with VF3. IEEE Trans Pattern Anal Mach Intell.

[24] Tsai W, Fu K (1979). Error-correcting isomorphisms of attributed relational graphs for pattern analysis. IEEE Trans Syst Man Cybern.

[25] Santacruz P, Serratosa F (2020). Error-tolerant graph matching in linear computational cost using an initial small partial matching. Pattern Recognit Lett..

[26] Bougleux S, Brun L, Carletti V, Foggia P, Gaüzère B, Vento M (2017). Graph edit distance as a quadratic assignment problem. Pattern Recognit Lett..

[27] Cortés X, Serratosa F (2015). Learning graph-matching edit-costs based on the optimality of the oracle’s node correspondences. Pattern Recognit Lett..

[28] Escolano F, Bonev B, Lozano MA, Jiang X, Ferrer M, Torsello A (2011). Information-geometric graph indexing from bags of partial node coverages. Graph-based representations in pattern recognition—8th IAPR-TC-15 international workshop, GbRPR 2011, Münster, Germany, May 18–20, 2011. Proceedings. Lecture notes in computer science.

[29] Kashima H, Tsuda K, Inokuchi A. Marginalized kernels between labeled graphs. In: Fawcett T, Mishra N, editprs. Machine learning, proceedings of the twentieth international conference (ICML 2003), August 21–24, 2003, Washington, DC, AAAI Press; 2003. p. 321–28. http://www.aaai.org/Library/ICML/2003/icml03-044.php

[30] Escolano F, Hancock ER, Lozano MA, Curado M (2017). The mutual information between graphs. Pattern Recognit Lett..

[31] Darwiche M, Conte D, Raveaux R, T’kindt V. Solving the graph edit distance problem with variable partitioning local search. In: Conte D, Ramel J, Foggia P, editors. Graph-based representations in pattern recognition—12th IAPR-TC-15 international workshop, GbRPR 2019, Tours, June 19–21, 2019, Proceedings. Lecture notes in computer science, vol 11510 (2019). p. 67–77. 10.1007/978-3-030-20081-7_7.

[32] Dwivedi SP, Singh RS (2018). Error-tolerant graph matching using node contraction. Pattern Recognit Lett..

[33] Lerouge J, Abu-Aisheh Z, Raveaux R, Héroux P, Adam S (2017). New binary linear programming formulation to compute the graph edit distance. Pattern Recognit..

[34] Riesen K, Bunke H (2009). Approximate graph edit distance computation by means of bipartite graph matching. Image Vis Comput.

[35] Serratosa F (2014). Fast computation of bipartite graph matching. Pattern Recognit Lett..

[36] Abu-Aisheh Z, Raveaux R, Ramel J (2016). Anytime graph matching. Pattern Recognit Lett..

[37] Jiang X, Bunke H (1999). Optimal quadratic-time isomorphism of ordered graphs. Pattern Recognit..

[38] Anari N, Vazirani VV (2020). Planar graph perfect matching is in NC. J ACM.

[39] Torsello A, Rowe DH, Pelillo M (2005). Polynomial-time metrics for attributed trees. IEEE Trans Pattern Anal Mach Intell.

[40] Fankhauser S, Riesen K, Bunke H, Jiang X, Ferrer M, Torsello A (2011). Speeding up graph edit distance computation through fast bipartite matching. Graph-based representations in pattern recognition—8th IAPR-TC-15 international workshop, GbRPR 2011, Münster, Germany, May 18–20, 2011. Proceedings. Lecture notes in computer science.

[41] Riesen K, Fischer A, Bunke H. Improved graph edit distance approximation with simulated annealing. In: Foggia P, Liu C, Vento M, editors. Graph-based representations in pattern recognition—11th IAPR-TC-15 international workshop, GbRPR 2017, Anacapri, Italy, May 16–18, 2017, Proceedings. Lecture notes in computer science, vol. 10310. 2017. p. 222–31. 10.1007/978-3-319-58961-9_20.

[42] Stauffer M, Tschachtli T, Fischer A, Riesen K. A survey on applications of bipartite graph edit distance. In: Foggia P, Liu C, Vento M, editors. Graph-based representations in pattern recognition—11th IAPR-TC-15 international workshop, GbRPR 2017, Anacapri, Italy, May 16–18, 2017, Proceedings. Lecture notes in computer science, vol. 10310. 2017. p. 242–52. 10.1007/978-3-319-58961-9_22.

[43] Riba P, Lladós J, Fornés A (2020). Hierarchical graphs for coarse-to-fine error tolerant matching. Pattern Recognit Lett..

[44] Mousavi SF, Safayani M, Mirzaei A, Bahonar H (2017). Hierarchical graph embedding in vector space by graph pyramid. Pattern Recognit..

[45] Dutta A, Riba P, Lladós J, Fornés A (2020). Hierarchical stochastic graphlet embedding for graph-based pattern recognition. Neural Comput Appl.

[46] Brin S, Page L (1998). The anatomy of a large-scale hypertextual web search engine. Comput Netw.

[47] Freeman LC (1977). A set of measures of centrality based on betweenness. Sociometry.

[48] Junior AU, Silveira RA, de Freitas Filho PJ, Uzinski JC, da Costa Bianchi RA. MASDES-DWMV: model for dynamic ensemble selection based on multiagent system and dynamic weighted majority voting. In: Martínez-Villaseñor L, Herrera-Alcántara O, Ponce HE, Castro-Espinoza F, editors. Advances in computational intelligence—19th Mexican international conference on artificial intelligence, MICAI 2020, Mexico City, Mexico, October 12–17, 2020, Proceedings, Part II. Lecture notes in computer science, vol. 12469. 2020. p. 419–34. 10.1007/978-3-030-60887-3_36.

[49] Eiben AE, Smith JE (2015). Introduction to evolutionary computing, natural computing series.

[50] Riesen K, Bunke H, da Vitoria Lobo N, Kasparis T, Roli F, Kwok JT, Georgiopoulos M, Anagnostopoulos GC, Loog M (2008). IAM graph database repository for graph based pattern recognition and machine learning. Structural, syntactic, and statistical pattern recognition, Joint IAPR international workshop, SSPR & SPR 2008, Orlando, USA, December 4–6, 2008. Proceedings. Lecture notes in computer science.

[51] Morris C, Kriege NM, Bause F, Kersting K, Mutzel P, Neumann M. Tudataset: a collection of benchmark datasets for learning with graphs. 2020. arXiv:2007.08663 [CoRR]

